# Modification of the Associations of Alcohol Intake With Serum Low-Density Lipoprotein Cholesterol and Triglycerides by *ALDH2* and *ADH1B* Polymorphisms in Japanese Men

**DOI:** 10.2188/jea.JE20160189

**Published:** 2018-04-05

**Authors:** Tae Sasakabe, Kenji Wakai, Sayo Kawai, Asahi Hishida, Mariko Naito, Sadao Suzuki, Yora Nindita, Kokichi Arisawa, Yoshikuni Kita, Megumi Hara, Nagato Kuriyama, Akie Hirata, Haruo Mikami, Isao Oze, Michiaki Kubo, Hideo Tanaka, Nobuyuki Hamajima

**Affiliations:** 1 Department of Preventive Medicine, Nagoya University Graduate School of Medicine, Nagoya, Japan; 2 Department of Public Health, Nagoya City University Graduate School of Medical Sciences, Nagoya, Japan; 3 Department of International Island and Community Medicine, Kagoshima University Graduate School of Medical and Dental Sciences, Kagoshima, Japan; 4 Department of Pharmacology and Therapeutic, Faculty of Medicine, Diponegoro University, Semarang, Indonesia; 5 Department of Preventive Medicine, Institute of Biomedical Sciences, Tokushima University Graduate School, Tokushima, Japan; 6 Faculty of Nursing Science, Tsuruga Nursing University, Tsuruga, Japan; 7 Department of Preventive Medicine, Faculty of Medicine, Saga University, Saga, Japan; 8 Department of Epidemiology for Community Health and Medicine, Kyoto Prefectural University of Medicine Graduate School of Medical Science, Kyoto, Japan; 9 Department of Geriatric Medicine, Graduate School of Medical Sciences, Kyushu University, Fukuoka, Japan; 10 Division of Cancer Registry, Prevention and Epidemiology, Chiba Cancer Center Research Institute, Chiba, Japan; 11 Division of Epidemiology and Prevention, Aichi Cancer Center Research Institute, Nagoya, Japan; 12 Center for Integrative Medical Sciences, RIKEN, Yokohama, Japan; 13 Department of Epidemiology, Nagoya University Graduate School of Medicine, Nagoya, Japan; 14 Department of Healthcare Administration, Nagoya University Graduate School of Medicine, Nagoya, Japan

**Keywords:** *ALDH2*, *ADH1B*, alcohol intake, lipid profile

## Abstract

**Background:**

Although beneficial associations have been reported between moderate alcohol intake and the serum lipid profile, it is unclear whether polymorphisms in alcohol-metabolizing enzymes can modify these associations. Here, we assessed the effects of *ADH1B* His48Arg (rs1229984), *ALDH2* Glu504Lys (rs671), and their combination on these associations. Furthermore, we examined if the findings for *ALDH2* could be replicated.

**Methods:**

We categorized 889 male participants in the Japan Multi-Institutional Collaborative Cohort (J-MICC) Study into two groups based on presence or absence of minor allele(s) or four groups based on genotype combinations. We performed regression analyses of serum lipid concentrations on alcohol intake, with multivariable adjustment. The replication study was conducted among 2,562 men in the Shizuoka part of the J-MICC Study.

**Results:**

The *ALDH2 Glu/Lys* or *Lys/Lys* groups showed significant decreases in serum low-density lipoprotein (LDL) cholesterol with increasing alcohol consumption; the coefficient per intake increase of 10 g/day was −2.49 mg/dL (95% confidence interval [CI], −3.85 to −1.13), and a significant interaction with the polymorphism was confirmed (*P* for interaction = 0.006). This inverse correlation was more evident among the *ADH1B His/His* + *ALDH2 Glu/Lys* or *Lys/Lys* groups (−3.24 mg/dL, 95% CI, −5.03 to −1.45). Serum triglycerides were positively associated with alcohol consumption in the *ADH1B His/His* group (*P* for interaction = 0.020). The stronger association between serum LDL cholesterol and alcohol consumption in the *ALDH2 Glu/Lys* or *Lys/Lys* groups was replicated.

**Conclusions:**

The *ALDH2* Glu504Lys polymorphism can modify the association between alcohol intake and serum LDL cholesterol in Japanese men.

## INTRODUCTION

Epidemiological evidence has shown that moderate alcohol consumption is negatively associated with the risk of coronary heart disease and stroke.^1^^–^^3^ This is partly explained by the beneficial effects of alcohol consumption on blood lipids, including an increase in high-density lipoprotein (HDL) cholesterol levels and a decrease in low-density lipoprotein (LDL) cholesterol levels. However, findings on the association between alcohol consumption and LDL cholesterol have been inconsistent.^4^ Furthermore, alcohol intake has also been associated with higher triglyceride levels.^5^^,^^6^ Although the molecular mechanisms underlying these associations remain under discussion, several regulatory steps in the process of lipid metabolism are thought to be involved.^7^^,^^8^ If so, the associations between alcohol consumption and blood lipids may be altered by alcohol metabolism-related gene variations that influence alcohol degradation; these gene variations have been described in studies of several diseases in Asian populations.^9^^,^^10^ Two enzymes, alcohol dehydrogenase (ADH) and aldehyde dehydrogenase (ALDH), play key roles in alcohol metabolism. Ethanol is oxidized to acetaldehyde by ADH and then metabolized into acetate by ALDH.^11^^,^^12^ Genetic polymorphisms in *ADH1B* (His48Arg) and *ALDH2* (Glu504Lys) have strong effects on alcohol metabolism. The rate of ethanol oxidation is accelerated in individuals carrying the *ADH1B His* allele, and over 70% of Asians have at least one *His* allele.^11^^,^^13^ In contrast, approximately 20% of Asians carry the *ALDH2 Lys* alleles.^14^ The *Lys* allele gives an inactive form of the enzyme, which leads to increased acetaldehyde levels after alcohol consumption due to a slower rate of acetaldehyde metabolism.^15^^,^^16^ There are almost no heterozygotes of these alcohol-related polymorphisms in Caucasian populations.^17^ Hence, the high activity of *ADH1B* and the low activity of *ALDH2* are rather specific to Asian populations.

Previous studies in Asian populations have focused mainly on the influence of the *ALDH2* genotype, and most studies have examined HDL cholesterol^18^^–^^22^; thus, the effects of this genotype on LDL cholesterol or triglycerides (TG) are still to be examined, and the combined effects of *ADH1B* and *ALDH2* polymorphisms on the associations between alcohol consumption and the lipid profile remain unclear. Therefore, the aim of this study was to evaluate the associations between alcohol intake and the serum lipid profile according to the genotypes of the alcohol-metabolizing enzymes *ADH1B* (His48Arg) and *ALDH2* (Glu504Lys) and their combinations in Japanese men, in order to gain insight into the association between moderate alcohol consumption and the risk of cardiovascular diseases.

## METHODS

### Study subjects

The study subjects were drawn from participants in the Japan Multi-Institutional Collaborative Cohort (J-MICC) Study.^23^ All subjects were aged between 35 and 69 years and had been voluntarily enrolled, mainly from health checkup examinees and the general population. Subjects provided blood samples and information on their lifestyles based on a questionnaire used between 2005 and 2008 in nine study areas, and in 2004 in one study area.^24^ The subjects in our cross-sectional study comprised approximately 500 participants consecutively recruited in each area, except for two areas where fewer participants had been recruited. We selected 4,490 participants at baseline in the J-MICC study. Written informed consent was obtained from all participants. This study was conducted in accordance with the Japanese government’s Ethical Guidelines for Human Genome and Genetic Analysis Research. The study protocol was approved by the ethics committees of Nagoya University Graduate School of Medicine and other participating institutions.

Of the male participants in seven areas with a >10-hr fasting serum lipid profile (*n* = 1,073), we excluded those without anthropometry (*n* = 1) or relevant genotypes (*n* = 1), and those who reported a history of hepatitis B or C, liver cirrhosis, liver cancer, or alcoholism (*n* = 51). We also excluded those with TG ≥1,000 mg/dL (*n* = 1) and those taking anti-diabetic or lipid-lowering drugs (*n* = 130). Ultimately, 889 subjects qualified for our analysis. However, participants receiving antihypertensive drugs were excluded from the blood pressure analyses (*n* = 151), while those with TG levels ≥400 mg/dL were excluded from the LDL cholesterol analyses (*n* = 17). We did not include female participants due to their very low alcohol intake.

### Lifestyle measurements

Lifestyle information was obtained using a common, standard self-administered questionnaire in all of the seven study areas. Alcohol consumption was estimated from the frequency of use (almost none, 1 to 3 days/month, 1 to 2 days/week, 3 to 4 days/week, 5 to 6 days/week, or everyday) and the amount consumed by beverage type. Participants reported consumption for each of the beverages consumed during the reported drinking session using prescribed portion sizes (eg, Japanese sake: 1 gou [180 mL], shochu: 1 gou [180 mL], shochu-based highball: 180 mL, beer: 633 mL, 500 mL, 350 mL and 250 mL, whiskey: 30 mL and 60 mL, and wine: 100 mL). Dietary intakes of energy, saturated fatty acids (SFA), polyunsaturated fatty acids (PUFA), cholesterol, and dietary fiber were estimated with a food frequency questionnaire containing 47 food items.^25^ Body mass index (BMI) was calculated using the following formula: body weight (kg)/(height [m])^2^. Physical activity in metabolic equivalents (METs) was estimated based on the frequency and intensity of daily activities and habitual exercise. The presence of diabetes was defined as a fasting blood glucose level ≥126 mg/dL or a hemoglobin A1c level ≥6.5% (values established by the National Glycohemoglobin Standardization Program).

### Measurement of serum lipid levels

Serum concentrations of total and HDL cholesterol and TG were measured as a part of the health checkups or for research purposes at the institutions participating in the J-MICC study. For the health examinations, it was not possible to directly control the procedures in most cases, as the majority of the data was collected during routine health checkups performed at other institutions. Measurements of the TG, total cholesterol, and HDL cholesterol were performed under the standardization program of the Japan Medical Association and/or the Center for Disease Control and Prevention in all of the study areas. However, we cannot rule out the possibility that there might have been some inter-institutional differences in these measurements. Therefore, as described in our statistical analysis section, we adjusted for these study areas in order to minimize the effect of these potential differences in the regression analyses. LDL cholesterol values were estimated using the Friedewald formula (LDL cholesterol = total cholesterol − HDL cholesterol − TG/5).

### Genotyping of polymorphisms

DNA was extracted from the buffy coat fractions using a BioRobot M48 Workstation (Qiagen Group, Tokyo, Japan) or from whole blood using an automatic nucleic acid isolation system (NA-3000; Kurabo, Co., Ltd., Osaka, Japan). The genotyping of *ADH1B* His48Arg (rs1229984) and *ALDH2* Glu504Lys (rs671) polymorphisms was conducted at the Laboratory for Genotyping Development, RIKEN Center for Genomic Medicine (Yokohama, Japan), using a multiplex polymerase chain reaction-based Invader assay (Third Wave Technologies, Madison, WI, USA).^26^

### Statistical analysis

Accordance with the Hardy-Weinberg equilibrium, which indicates the absence of discrepancy between genotypes and allele frequencies, was checked using the *χ*^2^ test. Serum TG and HDL cholesterol levels and intakes of SFA and cholesterol were log_e_-transformed to approximate a normal distribution. To categorize drinking habits during the analysis of the background characteristics, we separated participants into two groups (nondrinkers vs current drinkers) with former drinkers (*n* = 25) included in the nondrinkers group. We compared background characteristics between the genotypes or between the groups with and without minor allele(s). Continuous variables were tested with Student’s *t*-test, and categorical variables were assessed using the *χ*^2^ test in order to evaluate the differences between nondrinkers and current drinkers or between genotypes with or without the minor allele(s).

Associations between alcohol intake and the serum lipid profile were assessed by performing multiple regression analyses that incorporated alcohol consumption (continuous, g/day) as an independent variable and serum lipid concentrations as dependent variables. We also assessed the associations between the lipid concentrations and the drinking categories (non-drinkers [0 g], light drinkers [0.1–22.9 g], moderate drinkers [23.0–45.9 g], and heavy drinkers [≥46 g]). To avoid effects by potential confounders, we adjusted our analyses for age (continuous, years), physical activity (continuous, MET·hr/day), BMI (continuous, kg/m^2^), energy intake (continuous, kcal/day), diabetes (categorical, yes/no), education (categorical, over high school/high school or under), study area (categorical, six areas; with one area merged into the nearest area due to the small sample size), and smoking status (categorical, current smokers/former smokers/never smokers). The education level was included as an indicator of the socioeconomic status. We further adjusted for the intakes of SFA (continuous, g/day), PUFA (continuous, g/day), cholesterol (continuous, mg/day), and dietary fiber (continuous, g/day) in additional analyses.

We stratified the subjects into two groups (those with at least one minor allele and those without) or four groups (from the combinations of the two genotypes) in order to evaluate the effects of the polymorphisms on the associations between alcohol intake and the lipid profile. We determined the *P* values for interactions with the multiplicative terms calculated from the alcohol intake amount (continuous, 10 g/day) or the drinking categories (non-drinkers: 0, light drinkers: 1, moderate drinkers: 2, and heavy drinkers: 3) and the presence of each genotype group or combination group. The group or the combination with the homozygotes of the major alleles was set as the reference. For sensitivity analyses, we examined associations after excluding the participants in the top 25% of the alcohol consumption group, ie, those who consumed ≥39.1 g/day. Statistical analyses were conducted using Stata (ver. 13.1; Stata Corp., College Station, TX, USA). All *P* values were two-sided, and *P* values <0.05 were considered to be statistically significant.

### Replication study

We also examined whether the modifying effect exerted by the *ALDH2* polymorphism on the association between alcohol consumption and serum lipids in our main study could be replicated using existing data from an independent sample of 2,993 Japanese men aged 35 to 69 years from the Shizuoka part of the J-MICC study. Subjects were recruited from male examinees who visited a health checkup center in Hamamatsu, Shizuoka, Japan, between January 2006 and December 2007. After applying the same exclusion criteria used in the main study, 2,562 subjects were eligible for the replication analyses. We collected lifestyle information, measured serum lipid levels, and performed statistical analyses with the same methods as in the main study. The DigiTag assay was used to determine *ALDH2* genotype.^27^^,^^28^ All participants gave their written informed consent. The Shizuoka part of the J-MICC study was also conducted in accordance with the Japanese government’s Ethical Guidelines for Human Genome and Genetic Analysis Research. The study protocol was approved by the ethics committee of Nagoya University Graduate School of Medicine.

## RESULTS

### Participant characteristics

Table 1 shows the background characteristics of the participants, including the distributions of the *ADH1B* and *ALDH2* genotypes according to drinking habits. The average age of all participants included in the analyses was 55.1 (standard deviation [SD], 9.0) years. Among these participants, 678 were current drinkers (76%), and their average daily alcohol intake was 32.9 (SD, 28.3) g. The serum HDL cholesterol level was higher (*P* < 0.001) and the LDL cholesterol level was lower (*P* < 0.001) in the current drinkers than in the nondrinkers. The frequencies of both the *ADH1B* and *ALDH2* genotypes were in Hardy-Weinberg equilibrium (*P* = 0.99 and *P* = 0.60, respectively). The distribution of the *ALDH2* genotype greatly varied in accordance with the drinking habits; the *ALDH2 Glu/Lys* or *Lys/Lys* genotypes were present in 34.7% of the current drinkers versus in 81.5% of the nondrinkers.

**Table 1.** Background characteristics according to alcohol drinking habits in the main study^a^

	All (*n* = 889)	Nondrinkers^b^ (*n* = 211)	Current (*n* = 678)	*P*^c^
Age, years	55.1 (9.0)	55.8 (9.6)	54.9 (8.7)	0.186
Body mass index, kg/m^2^	23.7 (3.1)	23.7 (3.1)	23.7 (3.1)	0.767
Smoking				
Current smokers, %	27.8	25.6	28.5	0.147
Former smokers, %	43.0	39.8	44.0	
Nonsmokers, %	29.3	34.6	27.6	
Diabetes, %^d^	4.4	4.4	4.3	0.921
Education (over high school), %	44.9	44.4	46.5	0.601
Physical activity, MET·hr/day	14.2 (13.6)	15.3 (14.2)	13.9 (13.4)	0.192
Alcohol consumption, g/day	25.1 (28.4)	—	32.9 (28.3)	—
Systolic blood pressure, mm Hg^e^	123.5 (16.0)	121.6 (16.9)	124.1 (15.6)	0.064
Diastolic blood pressure, mm Hg^e^	77.8 (11.0)	76.1 (12.1)	78.4 (10.6)	0.015
Serum total cholesterol, mg/dL	205.4 (31.0)	206.1 (30.8)	205.2 (31.1)	0.722
Serum triglycerides, mg/dL^f^	128.6 (78.8)	122.2 (66.7)	130.6 (82.1)	0.257
Serum HDL cholesterol, mg/dL^f^	59.3 (15.9)	55.0 (14.4)	60.7 (16.1)	<0.001
Serum LDL cholesterol, mg/dL^g^	121.1 (29.3)	127.1 (28.3)	119.3 (29.3)	<0.001
Dietary intakes				
Energy, kcal/day	1923 (351)	1882 (332)	1936 (356)	0.053
Saturated fatty acids, g/day^f^	10.7 (2.4)	10.8 (2.6)	10.6 (2.3)	0.382
Polyunsaturated fatty acids, g/day	12.9 (3.4)	12.5 (3.5)	13.0 (3.3)	0.079
Cholesterol, mg/day^f^	235.1 (70.1)	226.2 (69.3)	237.8 (70.2)	0.018
Dietary fiber, g/day	9.9 (2.8)	10.2 (3.1)	9.9 (2.7)	0.163

*ADH1B* (rs1229984)				
*His/His*, %	58.2	59.7	57.7	0.062
*His/Arg*, %	36.2	37.9	35.7	
*Arg/Arg*, %	5.6	2.4	6.6	
*His/Arg* or *Arg/Arg*, %	41.8	40.3	42.3	
*ALDH2* (rs671)				
*Glu/Glu*, %	54.2	18.5	65.3	<0.001
*Glu/Lys*, %	38.4	51.7	34.2	
*Lys/Lys*, %	7.4	29.9	0.4	
*Glu/Lys* or *Lys/Lys*, %	45.8	81.5	34.7	

HDL, high-density lipoprotein; LDL, low-density lipoprotein; MET, metabolic equivalent.

^a^Values represent the means (standard deviation).

^b^Includes former drinkers (*n* = 25).

^c^Determined by *t*-test or chi-square test for comparison between nondrinkers and current drinkers.

^d^The presence of diabetes was defined as fasting blood glucose ≥126 mg/dL or hemoglobin A1c ≥6.5% (values of the National Glycohemoglobin Standardization Program).

^e^Participants receiving antihypertensive drugs were excluded from the analysis for blood pressure (*n* = 151).

^f^Statistically tested for log_e_-transformed values.

^g^Participants with serum triglycerides ≥400 mg/dL were excluded from the analysis for LDL cholesterol (*n* = 17).

When we compared background characteristics between the *ADH1B* and *ALDH2* genotypes (Table 2), a significant difference in BMI was found among the *ADH1B* genotypes. In contrast, age and LDL cholesterol were significantly lower, while BMI, alcohol consumption, systolic and diastolic blood pressure, serum TG, and HDL cholesterol were significantly higher in the participants with *ALDH2 Glu/Glu* when compared to those with *ALDH2 Glu/Lys* or *Lys/Lys*.

**Table 2.** Background characteristics according to the genotypes of *ADH1B* and *ALDH2* in the main study^a^

	*ADH1B* (rs1229984)					*ALDH2* (rs671)				
	
	*His/His*	*His/Arg*	*Arg/Arg*	*His/Arg* or *Arg/Arg*	*P*^b^	*Glu/Glu*	*Glu/Lys*	*Lys/Lys*	*Glu/Lys* or *Lys/Lys*	*P*^b^
*n*	517	322	50	372		482	341	66	407	
Age, years	54.8 (9.1)	55.5 (8.8)	55.7 (8.3)	55.5 (8.7)	0.220	54.5 (8.8)	55.7 (9.1)	56.4 (9.7)	55.8 (9.2)	0.032
Body mass index, kg/m^2^	23.5 (3.1)	23.9 (3.1)	24.4 (3.1)	23.9 (3.1)	0.030	23.9 (3.2)	23.5 (3.1)	23.1 (2.6)	23.4 (3.0)	0.012
Smoking										
Current smokers, %	27.5	28.6	26.0	28.2	0.955	27.4	29.6	21.2	28.3	0.068
Former smokers, %	43.7	41.3	46.0	41.9		40.3	45.8	48.5	46.2	
Nonsmokers, %	28.8	30.1	28.0	29.8		32.4	24.6	30.3	25.6	
Diabetes, %	4.8	4.4	0.0	3.8	0.441	5.0	3.5	4.6	3.7	0.348
Education (over high school), %	43.5	48.5	36.0	46.8	0.336	42.7	45.5	57.6	47.4	0.162
Physical activity, MET·hr/day	14.7 (14.2)	13.1 (12.4)	15.7 (15.0)	13.4 (12.8)	0.157	14.5 (14.1)	13.4 (12.8)	15.9 (14.2)	13.8 (13.1)	0.453
Alcohol consumption, g/day	24.3 (27.8)	25.6 (29.4)	30.4 (27.4)	26.2 (29.1)	0.311	35.1 (30.0)	15.7 (22.0)	0.3 (1.7)	13.2 (20.9)	<0.001
Systolic blood pressure, mm Hg^c^	123.9 (15.3)	123.0 (17.1)	122.8 (16.5)	123.0 (17.0)	0.455	125.3 (16.5)	121.8 (15.1)	120.0 (15.9)	121.5 (15.2)	0.001
Diastolic blood pressure, mm Hg^c^	77.7 (10.6)	78.0 (11.4)	77.5 (13.1)	77.9 (11.7)	0.807	79.2 (11.1)	76.3 (10.9)	75.9 (10.2)	76.3 (10.8)	<0.001
Serum total cholesterol, mg/dL	204.5 (31.5)	206.4 (30.1)	208.5 (32.6)	206.6 (30.4)	0.307	205.4 (31.7)	204.2 (30.1)	211.7 (30.2)	205.4 (30.2)	0.996
Serum triglycerides, mg/dL^d^	126.4 (76.9)	132.8 (84.5)	124.7 (56.8)	131.7 (81.3)	0.318	135.2 (86.1)	121.5 (69.0)	117.0 (65.5)	120.8 (68.4)	0.009
Serum HDL cholesterol, mg/dL^d^	60.1 (16.2)	58.4 (15.6)	57.6 (13.4)	58.3 (15.3)	0.104	60.5 (16.5)	58.4 (15.1)	55.3 (14.0)	57.9 (15.0)	0.016
Serum LDL cholesterol, mg/dL^e^	119.9 (29.4)	122.4 (29.2)	125.9 (27.9)	122.9 (29.0)	0.136	118.8 (30.2)	121.9 (27.6)	134.3 (27.3)	123.9 (27.9)	0.010
Dietary intakes										
Energy, kcal/day	1915 (343)	1933 (349)	1941 (447)	1934 (363)	0.432	1927 (360)	1917 (349)	1925 (301)	1918 (341)	0.724
Saturated fatty acids, g/day^d^	10.7 (2.4)	10.8 (2.5)	10.1 (1.9)	10.7 (2.4)	0.924	10.7 (2.4)	10.7 (2.4)	10.9 (2.3)	10.7 (2.4)	0.735
Polyunsaturated fatty acids, g/day	12.9 (3.4)	12.8 (3.3)	13.2 (3.4)	12.9 (3.3)	0.716	13.0 (3.2)	12.8 (3.7)	12.7 (2.7)	12.8 (3.5)	0.517
Cholesterol, mg/day^d^	235.1 (70.5)	235.6 (69.8)	231.8 (69.8)	235.1 (69.7)	0.883	236.0 (70.4)	233.1 (67.8)	237.9 (80.5)	233.9 (69.9)	0.612
Dietary fiber, g/day	9.9 (2.8)	9.9 (2.7)	9.9 (3.1)	9.9 (2.8)	0.922	9.8 (2.7)	10.1 (2.9)	10.2 (2.7)	10.1 (2.9)	0.149

HDL, high-density lipoprotein; LDL, low-density lipoprotein; MET, metabolic equivalent.

^a^Values represent the means (standard deviation).

^b^Determined by *t*-test or chi-square test for comparison between genotypes with or without minor allele(s).

^c^Participants receiving antihypertensive drugs were excluded from the analysis for blood pressure (*n* = 151).

^d^Statistically tested by log_e_-transformed values.

^e^Participants with serum triglycerides ≥400 mg/dL were excluded from the analysis for LDL cholesterol (*n* = 17).

### Associations between alcohol intake and lipid profile according to genotype

**Figure 1.**
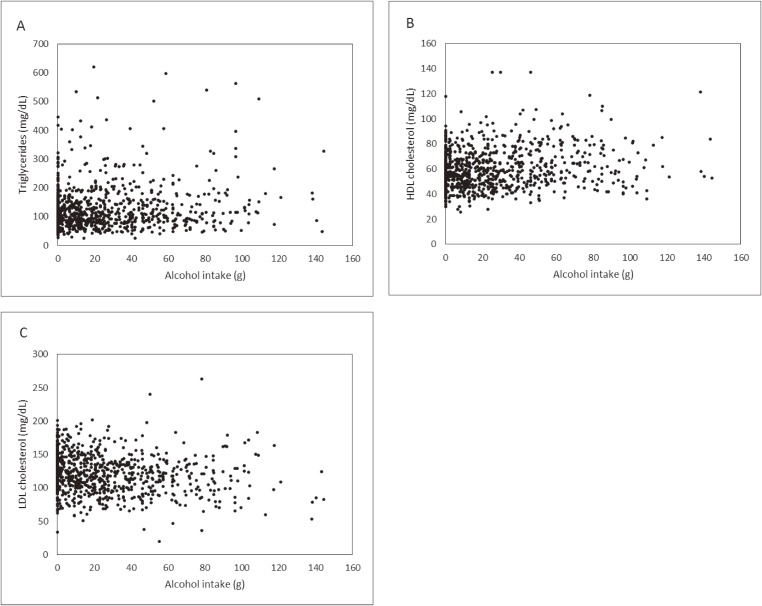
Scatter diagrams of alcohol intake and triglycerides (A), HDL cholesterol (B) and LDL cholesterol (C) in the main study. HDL, high-density lipoprotein; LDL, low-density lipoprotein.

The serum concentrations of HDL cholesterol and LDL cholesterol were significantly associated with alcohol intake (Table 3 and Figure 1); the HDL cholesterol level increased (β = 0.024; 95% confidence interval [CI], 0.019–0.030) and the LDL cholesterol level decreased (β = −1.47; 95% CI, −2.20 to −0.75) with increasing alcohol intake. In the assessment of each genotype, serum TG was positively and significantly correlated with alcohol consumption in the *ADH1B His/His* group (β = 0.017; 95% CI, 0.001–0.032), and a significant interaction was observed between this polymorphism and alcohol intake (*P* = 0.020). Meanwhile, the LDL cholesterol level in *ALDH2 Glu/Lys* or *Lys/Lys* participants significantly decreased with increasing alcohol intake (β = −2.49; 95% CI, −3.85 to −1.13), with a significant interaction between the polymorphism and alcohol intake (*P* = 0.006), while those in both *ADH1B* genotype groups showed a significant inverse correlation between alcohol consumption and LDL cholesterol level (β = −1.59; 95% CI, −2.56 to −0.62 for *ADH1B His/His* and β = −1.30; 95% CI, −2.40 to −0.19 for *ADH1B His/Arg* or *Arg/Arg*). The serum HDL cholesterol level increased with increasing alcohol consumption in every genotype group, and no interaction was observed between alcohol consumption and the different genotypes. In the analyses of the combination of *ADH1B* and *ALDH2* genotypes, a stronger inverse association between alcohol consumption and serum LDL cholesterol level was found in individuals with *ADH1B His/His* and *ALDH2 Glu/Lys* or *Lys/Lys* (β = −3.24; 95% CI, −5.03 to −1.45; *P* for interaction = 0.010; Table 4). This association remained after exclusion of the participants in the top 25% of the alcohol consumption group (ie, those who consumed ≥39.1 g/day; β = −5.25; 95% CI, −8.62 to −1.89 and *P* for interaction = 0.009; data not shown). The findings in Table 3 and Table 4 were not substantially changed after additional adjustments for the intakes of SFA, PUFA, cholesterol, and dietary fiber, although the adjusted coefficient for TG was not statistically significant in the *ADH1B His/His* group (eTable 1). When assessed according to the drinking level, the results were almost unchanged. Decreased LDL cholesterol levels were observed when the *ALDH2 Lys* allele (eTable 2) or the *ADH1B His/His* and *ALDH2 Glu/Lys* or *Lys/Lys* genotypes (eTable 3) were present in subjects with higher drinking levels. Interactions were also detected between the genotypes and alcohol consumption.

**Table 3.** Increase in serum lipid levels with an increase of alcohol intake by 10 g/day according to the genotypes of *ADH1B* and *ALDH2* in the main study^a^

	Log_e_-triglycerides (mg/dL)			Log_e_-HDL cholesterol (mg/dL)			LDL cholesterol (mg/dL)		
		
	*n*	β	(95% Cl)	*n*	β	(95% Cl)	*n*	β	(95% Cl)
All	889	0.006	(−0.006 to 0.018)	889	0.024	(0.019–0.030)	872	−1.47	(−2.20 to −0.75)
Genotypes									
*ADH1B* (rs1229984)									
*His/His*	517	0.017	(0.001–0.032)	517	0.024	(0.017–0.032)	508	−1.59	(−2.56 to −0.62)
*P* for interaction			reference			reference			reference
*His/Arg* or *Arg/Arg*	372	−0.005	(−0.024 to 0.014)	372	0.025	(0.016–0.033)	364	−1.30	(−2.40 to −0.19)
*P* for interaction			0.020			0.609			0.871
*ALDH2* (rs671)									
*Glu/Glu*	482	0.002	(−0.014 to 0.018)	482	0.025	(0.017–0.032)	470	−0.73	(−1.72 to 0.25)
*P* for interaction			reference			reference			reference
*Glu/Lys* or *Lys/Lys*	407	0.004	(−0.019 to 0.027)	407	0.021	(0.010–0.032)	402	−2.49	(−3.85 to −1.13)
*P* for interaction			0.772			0.667			0.006

CI, confidence interval; HDL, high-density lipoprotein; LDL, low-density lipoprotein.

^a^Determined by linear regression analysis adjusted for age, physical activity, body mass index, energy intake, study area, education, diabetes and smoking status as covariates.

**Table 4.** Increase in serum lipid levels with an increase of alcohol intake by 10 g/day according to the combinations of *ADH1B* and *ALDH2* genotypes in the main study^a^

	Log_e_-triglycerides (mg/dL)			Log_e_-HDL cholesterol (mg/dL)			LDL cholesterol (mg/dL)		
		
	*n*	β	(95% Cl)	*n*	β	(95% Cl)	*n*	β	(95% Cl)
Combinations of genotypes									
*ADH1B His/His* + *ALDH2 Glu/Glu*	275	0.010	(−0.010 to 0.030)	275	0.023	(0.013–0.032)	270	−0.52	(−1.85 to 0.80)
*P* for interaction			reference			reference			reference
*ADH1B His/His* + *ALDH2 Glu/Lys* or *Lys/Lys*	242	0.032	(0.0007–0.064)	242	0.020	(0.005–0.036)	238	−3.24	(−5.03 to −1.45)
*P* for interaction			0.473			0.713			0.010
*ADH1B His/Arg* or *Arg/Arg* + *ALDH2 Glu/Glu*	207	−0.011	(−0.038 to 0.017)	207	0.028	(0.016–0.040)	200	−0.97	(−2.50 to 0.55)
*P* for interaction			0.161			0.537			0.548
*ADH1B His/Arg* or *Arg/Arg* + *ALDH2 Glu/Lys* or *Lys/Lys*	165	−0.031	(−0.066 to 0.004)	165	0.020	(0.002–0.038)	164	−0.90	(−3.09 to 1.30)
*P* for interaction			0.017			0.890			0.109

CI, confidence interval; HDL, high-density lipoprotein; LDL, low-density lipoprotein.

^a^Determined by linear regression analysis adjusted for age, physical activity, body mass index, energy intake, study area, education, diabetes and smoking status as covariates.

### Associations between alcohol intake and lipid profile according to the *ALDH2* genotype in the replication study

In the replication study, the distribution of *ALDH2* genotypes was in Hardy-Weinberg equilibrium, and the minor allele frequency was 0.30. The average age of the replication study participants was 52.3 (SD, 8.8) years. Among these participants, 1,944 were current drinkers (76%), and their average daily alcohol intake was 25.9 (SD, 25.8) g (eTable 4).

In the replication study, alcohol consumption was significantly associated with the serum concentrations of HDL cholesterol and LDL cholesterol, in addition to serum TG. When we assessed whether the association between alcohol consumption and LDL cholesterol level was modified by *ALDH2* polymorphism, we largely replicated the modification found in the main study. The *ALDH2 Glu/Lys* or *Lys/Lys* groups showed a significantly decreased LDL cholesterol level with increasing alcohol consumption; the coefficient per intake increase of 10 g/day was −2.53 mg/dL (95% CI, −3.53 to −1.53). The *ALDH2 Glu/Glu* group also showed a significant association, but it was weaker than in the *Glu/Lys* or *Lys/Lys* groups (interaction *P* = 0.002; Table 5). When assessed according to the level of drinking, LDL cholesterol decreased in the subjects that had higher drinking levels and *ALDH2 Glu/Lys* or *Lys/Lys* as well, which was similar to the main study. However, an interaction between the genotypes and alcohol intake was not detected (eTable 5).

**Table 5.** Increase in serum lipid levels with an increase of alcohol intake by 10 g/day according to the genotypes of *ALDH2* in the replication study^a^

	Log_e_-triglycerides (mg/dL)			Log_e_-HDL cholesterol (mg/dL)			LDL cholesterol (mg/dL)		
		
	*n*	β	(95% Cl)	*n*	β	(95% Cl)	*n*	β	(95% Cl)
All	2562	0.008	(0.001–0.016)	2562	0.020	(0.016–0.024)	2531	−1.23	(−1.70 to −0.77)
Genotypes									
*ALDH2* (rs671)									
*Glu/Glu*	1253	0.009	(−0.001 to 0.018)	1253	0.013	(0.008–0.018)	1237	−0.79	(−1.38 to −0.19)
*P* for interaction			reference			reference			reference
*Glu/Lys* or *Lys/Lys*	1309	0.003	(−0.013 to 0.019)	1309	0.025	(0.018–0.033)	1294	−2.53	(−3.53 to −1.53)
*P* for interaction			0.221			0.004			0.002

CI, confidence interval; HDL, high-density lipoprotein; LDL, low-density lipoprotein.

^a^Determined by linear regression analysis adjusted for age, physical activity, body mass index, energy intake, education, diabetes and smoking status as covariates.

## DISCUSSION

When we examined the decrease in the serum LDL cholesterol level among Japanese men, we observed a significant interaction of alcohol intake with the *ALDH2 Glu/Lys* or *Lys/Lys* genotypes, as well as with the combination of *ADH1B His/His* and *ALDH2 Glu/Lys* or *Lys/Lys* genotypes. The positive correlation between serum TG and alcohol consumption was stronger in men with *ADH1B His/His* than in those with *ADH1B His/Arg* or *Arg/Arg*. Furthermore, we replicated the findings of our main study that demonstrated the *ALDH2* polymorphism modified the association between alcohol consumption and serum LDL cholesterol.

Prior studies have yielded conflicting results on the relationship between alcohol consumption and blood LDL cholesterol.^29^^–^^33^ Recently, it was proposed that these different observations may be connected to genetic variations in the metabolism of apolipoproteins, particularly the apolipoprotein A5 polymorphisms.^4^ Alternatively, the differences may be related to polymorphisms in alcohol-metabolizing genes. However, previous research that assessed the modifying effects of alcohol-related genes in Caucasians found almost no significant interactions between alcohol intake and the *ADH* or *ALDH* genetic polymorphisms on blood LDL cholesterol or non-HDL cholesterol levels.^5^^,^^34^^,^^35^ One study showed a significant interaction effect between alcohol intake and the *ADH1B His/His* or *His/Arg* genotype, which caused a decrease in the LDL cholesterol level; however, no interactions were observed with other *ADH* and *ALDH* gene polymorphisms.^35^ In Asians, one study demonstrated a possible association between the *ALDH2 Glu/Lys* or *Lys/Lys* genotype and a rather high LDL cholesterol level in the general population, after adjusting for drinking habits.^22^ However, other studies that adjusted for alcohol intake failed to detect any associations.^20^^,^^21^ In alcoholic men, a low odds ratio for a high LDL cholesterol level was reported in those with the *ADH1B His* allele.^36^ However, our study found a significant interaction between alcohol intake and the presence of the *ALDH2* minor allele (*Glu/Lys* or *Lys/Lys*), and an even stronger effect in the combination with *ADH1B His/His*. In our replication study, a similarly significant interaction was observed for the *ALDH2 Glu/Lys* polymorphism. Thus, inconsistencies between previous studies may be explained via differences in minor allele frequencies among populations, the inclusion of only moderate-to-heavy drinkers,^20^^,^^36^ or dissimilarities in statistical methodology. In our main study, 41.8% and 45.8% of the participants had at least one minor allele of the *ADH1B* and *ALDH2* polymorphisms, respectively. This enabled us to have sufficient power to examine the interactions.

Additionally, although the mean LDL cholesterol level was higher in subjects with *ALDH2 Glu/Lys or Lys/Lys* genotype compared to those with *ALDH2 Glu/Glu*, LDL cholesterol significantly decreased with increasing alcohol intake in the former group. This suggests that the beneficial effect of a moderate alcohol intake on LDL cholesterol is more apparent in those with *ALDH2 Glu/Lys* or *Lys/Lys*. Participants with *ALDH2 Glu/Glu* had higher HDL cholesterol and TG concentrations, which might lead a lower mean estimated LDL cholesterol.

Almost all studies have shown a robust relationship between alcohol intake and an increase in the TG or HDL cholesterol level; however, results have been inconsistent regarding the modifying effects of genetic variations, especially in alcohol metabolism-related genes.^4^ Although previous studies that examined Asian general populations have shown that the TG or HDL cholesterol level was significantly higher in alcohol drinkers with sufficient ALDH2 activity (as determined by the skin reaction test) compared to nondrinkers, interactions between lipid levels and the ALDH2 phenotype were either not assessed or not detected.^18^^,^^19^ In another study performed in alcoholics, the presence of the *ADH1B His* allele and *ALDH2 Glu/Glu* genotype was associated with the prevalence of hypertriglyceridemia or a lower HDL cholesterol level.^36^ Our main study showed that there was an increase in the TG level with increasing alcohol intake for the *ADH1B His/His* genotype, but not for each of the *ALDH2* genotypes. Since moderate alcohol consumption increases lipoprotein lipase activity, this may decrease the TG level and reduce the risk of fatty liver.^37^^,^^38^ However, we did not detect a decrease in the TG in relation to moderate alcohol drinking.

We also found that alcohol consumption was associated with an increase in the serum HDL cholesterol level regardless of the *ADH1B* or *ALDH2* genotype. Polymorphisms in other genes, such as those encoding apolipoproteins or cholesteryl ester transfer protein, might have a more pronounced effect than *ADH* and *ALDH*.^39^

From a biological point of view, the combination of a rapid first step in metabolizing alcohol to acetaldehyde with a longer exposure to acetaldehyde—a phenotype associated with the combination of *ADH1B His/His* and *ALDH2 Glu/Lys* or *Lys/Lys* genotypes—may partly explain the findings of the present study. Interestingly, one report indicated that blood acetaldehyde concentration after the intake of a moderate dose of ethanol appeared to be approximately six times greater in individuals with *ADH1B His/His* and *ALDH2 Glu/Lys* compared to those with *ADH1B His/His* and *ALDH2 Glu/Glu*, and approximately doubled when compared to those with *ADH1B His/Arg* and *ALDH2 Glu/Lys*.^40^ The formation of acetaldehyde adducts with apolipoprotein B may reduce the conversion of very low-density lipoprotein cholesterol to LDL cholesterol, which would decrease the serum LDL cholesterol level.^41^^–^^43^ Therefore, the varying levels of acetaldehyde derived from alcohol consumption might influence the serum LDL cholesterol level. Such effects of the *ADH1B* or *ALDH2* genotype may partly account for the inconsistencies in LDL cholesterol level changes that have been associated with alcohol consumption in different populations. Regarding the influence of alcohol on circulating TG, both ADH and ALDH use nicotinamide adenine dinucleotide (NAD)^+^, which is reduced to NADH, as a cofactor in both steps of alcohol metabolism. The increased production of NADH may disturb the tricarboxylic acid cycle and β-oxidation of fatty acids, thereby resulting in higher TG levels.^44^^,^^45^ Thus, genetic differences in the rate of alcohol degradation may alter NADH levels. The *ADH1B His* alleles (which are associated with fast metabolism) may increase NADH production more rapidly than the *ADH1B Arg* alleles (which are associated with slow metabolism), thereby increasing the serum TG level.

The aim of the present study was to assess the interaction between alcohol intake and genetic variations and its effect on the lipid profile in an Asian population with specific polymorphisms in alcohol metabolism-related genes. The strength of our study was that we replicated the modifying effect of the *ALDH2* polymorphism on the association between alcohol intake and serum LDL cholesterol in a larger, independent sample. However, there were several limitations. First, alcohol consumption was relatively lower in participants with the *ALDH2 Lys* alleles than in those with the *ALDH2 Glu/Glu* genotype because of their slower acetaldehyde metabolism.^15^^,^^16^ Therefore, the influence of heavy alcohol drinking might be difficult to assess in those with the *ALDH2 Lys* alleles. However, our findings may be indicative of the influence of moderate or lower alcohol intake because they still showed significance after we excluded the participants in the top 25% of the alcohol consumption group (ie, those who consumed ≥39.1 g/day). Second, in consideration of the age range utilized in the J-MICC Study and the fact that there is a higher intake of alcohol in men versus women, only middle-aged men (35–69 years) were included in this study. Therefore, evaluations in females and older participants will also need to be performed in order to confirm these results. Third, since we used pre-existing data, we could not examine the modifying effect of the *ADH1B* polymorphism in our replication study. It would be valuable to assess the influence of the *ALDH2* polymorphism on blood lipids, because the impact may be considerable, especially in Asian populations.

We would caution against alcohol consumption by individuals with the *ALDH2 Glu/Lys* or *Lys/Lys* genotype because of other health concerns, such as the higher risk of alcohol-related cancer.^9^ However, our current findings imply that genetic modifications might be indicative of the possible beneficial effects of moderate alcohol intake on the risk of cardiovascular diseases. Our current findings also suggest that these polymorphisms could have quite strong impacts on the various aspects of metabolism and human health. We believe that our study could contribute to the cumulative evidence for the prevention of lifestyle-related diseases considering to the gene-environmental interactions.

In summary, the *ALDH2* Glu504Lys polymorphism modified the association between alcohol intake and serum LDL cholesterol. This association was even stronger with the *ALDH2 Glu/Lys* or *Lys/Lys* genotype, which lessens the ability to metabolize alcohol. Further studies on polymorphisms related to alcohol metabolism or on the long-term effects of the *ALDH2* genotype are warranted, especially in Asian populations, where the minor alleles are relatively common.

## APPENDIX A. SUPPLEMENTARY DATA

eTable 1. Increase in serum lipid levels with an increase of alcohol intake by 10 g/day according to the *ADH1B* and *ALDH2* genotypes and their combinations (with additional adjustment for dietary intakes) in the main study^a^

eTable 2. Associations between serum lipid levels and drinking categories according to the presence of *ADH1B* and *ALDH2* in the main study^a^

eTable 3. Associations between serum lipid levels and drinking categories according to the combination of *ADH1B* and *ALDH2* genotypes in the main study^a^

eTable 4. Background characteristics according to alcohol drinking habits in the replication study^a^

eTable 5. Associations between serum lipid levels and drinking categories in the replication study^a^

## References

[1] Ronksley PE, Brien SE, Turner BJ, Mukamal KJ, Ghali WA Association of alcohol consumption with selected cardiovascular disease outcomes: a systematic review and meta-analysis. BMJ. 2011;342:d671. 10.1136/bmj.d671 21343207 PMC3043109

[2] Reynolds K, Lewis B, Nolen JD, Kinney GL, Sathya B, He J Alcohol consumption and risk of stroke: a meta-analysis. JAMA. 2003;289:579–588. 10.1001/jama.289.5.579 12578491

[3] Iso H, Kitamura A, Shimamoto T, Alcohol intake and the risk of cardiovascular disease in middle-aged Japanese men. Stroke. 1995;26:767–773. 10.1161/01.STR.26.5.767 7740564

[4] Brinton EA Effects of ethanol intake on lipoproteins. Curr Atheroscler Rep. 2012;14:108–114. 10.1007/s11883-012-0230-7 22350634

[5] Tolstrup JS, Grønbaek M, Nordestgaard BG Alcohol intake, myocardial infarction, biochemical risk factors, and alcohol dehydrogenase genotypes. Circ Cardiovasc Genet. 2009;2:507–514. 10.1161/CIRCGENETICS.109.873604 20031627

[6] Klop B, do Rego AT, Cabezas MC Alcohol and plasma triglycerides. Curr Opin Lipidol. 2013;24:321–326. 10.1097/MOL.0b013e3283606845 23511381

[7] Schneider J, Liesenfeld A, Mordasini R, Lipoprotein fractions, lipoprotein lipase and hepatic triglyceride lipase during short-term and long-term uptake of ethanol in healthy subjects. Atherosclerosis. 1985;57:281–291. 10.1016/0021-9150(85)90040-1 4084359

[8] Nishiwaki M, Ishikawa T, Ito T, Effects of alcohol on lipoprotein lipase, hepatic lipase, cholesteryl ester transfer protein, and lecithin:cholesterol acyltransferase in high-density lipoprotein cholesterol elevation. Atherosclerosis. 1994;111:99–109. 10.1016/0021-9150(94)90195-3 7840818

[9] Oze I, Matsuo K, Wakai K, ; Research Group for the Development and Evaluation of Cancer Prevention Strategies in Japan Alcohol drinking and esophageal cancer risk: an evaluation based on a systematic review of epidemiologic evidence among the Japanese population. Jpn J Clin Oncol. 2011;41:677–692. 10.1093/jjco/hyr026 21430021

[10] Kanda J, Matsuo K, Suzuki T, Impact of alcohol consumption with polymorphisms in alcohol-metabolizing enzymes on pancreatic cancer risk in Japanese. Cancer Sci. 2009;100:296–302. 10.1111/j.1349-7006.2008.01044.x 19068087 PMC11159673

[11] Bosron WF, Li TK Genetic polymorphism of human liver alcohol and aldehyde dehydrogenases, and their relationship to alcohol metabolism and alcoholism. Hepatology. 1986;6:502–510. 10.1002/hep.1840060330 3519419

[12] Seitz HK, Stickel F Molecular mechanisms of alcohol-mediated carcinogenesis. Nat Rev Cancer. 2007;7:599–612. 10.1038/nrc2191 17646865

[13] National Center for Biotechnology Information. Available at: http://www.ncbi.nlm.nih.gov/SNP/snp_ref.cgi?rs=1229984. Accessed: 10 April 2015.

[14] National Center for Biotechnology Information. Available at: http://www.ncbi.nlm.nih.gov/SNP/snp_ref.cgi?rs=671. Accessed: 10 April 2015.

[15] Yoshida A, Huang IY, Ikawa M Molecular abnormality of an inactive aldehyde dehydrogenase variant commonly found in Orientals. Proc Natl Acad Sci USA. 1984;81:258–261. 10.1073/pnas.81.1.258 6582480 PMC344651

[16] Hempel J, Kaiser R, Jörnvall H Human liver mitochondrial aldehyde dehydrogenase: a C-terminal segment positions and defines the structure corresponding to the one reported to differ in the Oriental enzyme variant. FEBS Lett. 1984;173:367–373. 10.1016/0014-5793(84)80807-8 6745443

[17] Brennan P, Lewis S, Hashibe M, Pooled analysis of alcohol dehydrogenase genotypes and head and neck cancer: a HuGE review. Am J Epidemiol. 2004;159:1–16. 10.1093/aje/kwh003 14693654

[18] Nakamura S, Ito Y, Suzuki K, Hashimoto S Blood pressure, levels of serum lipids, liver enzymes and blood glucose by aldehyde dehydrogenase 2 and drinking habit in Japanese men. Environ Health Prev Med. 2006;11:82–88. 10.1007/BF02898147 21432367 PMC2723637

[19] Nakamura Y, Amamoto K, Tamaki S, Genetic variation in aldehyde dehydrogenase 2 and the effect of alcohol consumption on cholesterol levels. Atherosclerosis. 2002;164:171–177. 10.1016/S0021-9150(02)00059-X 12119207

[20] Hashimoto Y, Nakayama T, Futamura A, Omura M, Nakarai H, Nakahara K Relationship between genetic polymorphisms of alcohol-metabolizing enzymes and changes in risk factors for coronary heart disease associated with alcohol consumption. Clin Chem. 2002;48:1043–1048. 12089173

[21] Takeuchi F, Isono M, Nabika T, Confirmation of ALDH2 as a Major locus of drinking behavior and of its variants regulating multiple metabolic phenotypes in a Japanese population. Circ J. 2011;75:911–918. 10.1253/circj.CJ-10-0774 21372407

[22] Kotani K, Sakane N, Yamada T Association of an aldehyde dehydrogenase 2 (ALDH2) gene polymorphism with hyper-low-density lipoprotein cholesterolemia in a Japanese population. Ethn Dis. 2012;22:324–328. 22870576

[23] Hamajima N; J-MICC Study Group The Japan Multi-Institutional Collaborative Cohort Study (J-MICC Study) to detect gene-environment interactions for cancer. Asian Pac J Cancer Prev. 2007;8:317–323. 17696755

[24] Wakai K, Hamajima N, Okada R, ; J-MICC Study Group Profile of participants and genotype distributions of 108 polymorphisms in a cross-sectional study of associations of genotypes with lifestyle and clinical factors: a project in the Japan Multi-Institutional Collaborative Cohort (J-MICC) Study. J Epidemiol. 2011;21:223–235. 10.2188/jea.JE20100139 21467728 PMC3899413

[25] Tokudome Y, Goto C, Imaeda N, Relative validity of a short food frequency questionnaire for assessing nutrient intake versus three-day weighed diet records in middle-aged Japanese. J Epidemiol. 2005;15:135–145. 10.2188/jea.15.135 16141632 PMC7851066

[26] Ohnishi Y, Tanaka T, Ozaki K, Yamada R, Suzuki H, Nakamura Y A high-throughput SNP typing system for genome-wide association studies. J Hum Genet. 2001;46:471–477. 10.1007/s100380170047 11501945

[27] Nishida N, Tanabe T, Hashido K, DigiTag assay for multiplex single nucleotide polymorphism typing with high success rate. Anal Biochem. 2005;346:281–288. 10.1016/j.ab.2005.08.007 16185645

[28] Nishida N, Tanabe T, Takasu M, Suyama A, Tokunaga K Further development of multiplex single nucleotide polymorphism typing method, the DigiTag2 assay. Anal Biochem. 2007;364:78–85. 10.1016/j.ab.2007.02.005 17359929

[29] Wakabayashi I, Groschner K Modification of the association between alcohol drinking and non-HDL cholesterol by gender. Clin Chim Acta. 2009;404:154–159. 10.1016/j.cca.2009.03.047 19336233

[30] Wakabayashi I, Araki Y Associations of alcohol consumption with blood pressure and serum lipids in Japanese female smokers and nonsmokers. Gend Med. 2009;6:290–299. 10.1016/j.genm.2009.04.005 19467525

[31] Wakabayashi I, Groschner K Age-dependent associations of smoking and drinking with non-high-density lipoprotein cholesterol. Metabolism. 2010;59:1074–1081. 10.1016/j.metabol.2009.11.004 20045152

[32] Perissinotto E, Buja A, Maggi S, ; ILSA Working Group Alcohol consumption and cardiovascular risk factors in older lifelong wine drinkers: the Italian Longitudinal Study on Aging. Nutr Metab Cardiovasc Dis. 2010;20:647–655. 10.1016/j.numecd.2009.05.014 19695851

[33] Onat A, Hergenc G, Dursunoglu D, Associations of alcohol consumption with blood pressure, lipoproteins, and subclinical inflammation among Turks. Alcohol. 2008;42:593–601. 10.1016/j.alcohol.2008.06.007 18835594

[34] Lawlor DA, Nordestgaard BG, Benn M, Zuccolo L, Tybjaerg-Hansen A, Davey Smith G Exploring causal associations between alcohol and coronary heart disease risk factors: findings from a Mendelian randomization study in the Copenhagen General Population Study. Eur Heart J. 2013;34:2519–2528. 10.1093/eurheartj/eht081 23492672

[35] Husemoen LL, Jørgensen T, Borch-Johnsen K, Hansen T, Pedersen O, Linneberg A The association of alcohol and alcohol metabolizing gene variants with diabetes and coronary heart disease risk factors in a white population. PLoS One. 2010;5:e11735. 10.1371/journal.pone.0011735 20700531 PMC2916825

[36] Yokoyama A, Yokoyama T, Matsui T, Alcohol dehydrogenase-1B (rs1229984) and aldehyde dehydrogenase-2 (rs671) genotypes are strong determinants of the serum triglyceride and cholesterol levels of Japanese alcoholic men. PLoS One. 2015;10:e0133460. 10.1371/journal.pone.0133460 26284938 PMC4540432

[37] Hashimoto Y, Hamaguchi M, Kojima T, Modest alcohol consumption reduces the incidence of fatty liver in men: a population-based large-scale cohort study. J Gastroenterol Hepatol. 2015;30:546–552. 10.1111/jgh.12786 25238605

[38] Kovář J, Zemánková K Moderate alcohol consumption and triglyceridemia. Physiol Res. 2015;64(Suppl 3):S371–S375. 26680670 10.33549/physiolres.933178

[39] Jensen MK, Mukamal KJ, Overvad K, Rimm EB Alcohol consumption, TaqIB polymorphism of cholesteryl ester transfer protein, high-density lipoprotein cholesterol, and risk of coronary heart disease in men and women. Eur Heart J. 2008;29:104–112. 10.1093/eurheartj/ehm517 18063597

[40] Kang G, Bae KY, Kim SW, Effect of the allelic variant of alcohol dehydrogenase ADH1B^*^2 on ethanol metabolism. Alcohol Clin Exp Res. 2014;38:1502–1509. 10.1111/acer.12427 24797321

[41] Kesäniemi YA, Kervinen K, Miettinen TA Acetaldehyde modification of low density lipoprotein accelerates its catabolism in man. Eur J Clin Invest. 1987;17:29–36. 10.1111/j.1365-2362.1987.tb01222.x 3106048

[42] Wehr H, Rodo M, Lieber CS, Baraona E Acetaldehyde adducts and autoantibodies against VLDL and LDL in alcoholics. J Lipid Res. 1993;34:1237–1244. 8371070

[43] Savolainen MJ, Baraona E, Lieber CS Acetaldehyde binding increases the catabolism of rat serum low-density lipoproteins. Life Sci. 1987;40:841–846. 10.1016/0024-3205(87)90032-4 3821381

[44] Lieber CS Alcoholic fatty liver: its pathogenesis and mechanism of progression to inflammation and fibrosis. Alcohol. 2004;34:9–19. 10.1016/j.alcohol.2004.07.008 15670660

[45] Purohit V, Gao B, Song BJ Molecular mechanisms of alcoholic fatty liver. Alcohol Clin Exp Res. 2009;33:191–205. 10.1111/j.1530-0277.2008.00827.x 19032584 PMC2633431

