# Supplementary material for: Modification of the Associations of Alcohol Intake With Serum Low-Density Lipoprotein Cholesterol and Triglycerides by ALDH2 and ADH1B Polymorphisms in Japanese Men
Source: J Epidemiol. 2018 Apr 5;28(4):185–93. doi: 10.2188/jea.JE20160189 (PMC5865009; doi:10.2188/jea.JE20160189)
Supplement: Supplementary file 1 [file je-28-185-s001.pdf]

eTable 1. Increase in serum lipid levels with an increase of alcohol intake by 10 g/day according to the *ADH1B* and *ALDH2* genotypes and their combinations (with additional adjustment for dietary intakes) in the main study[a]

	$\log_e$-triglycerides (mg/dL)			$\log_e$-HDL cholesterol (mg/dL)			LDL cholesterol (mg/dL)		
	n	β	(95% Cl)	n	β	(95% Cl)	n	β	(95% Cl)
All	889	0.006	(−0.006 to 0.018)	889	0.024	(0.019–0.030)	872	−1.47	(−2.15 to −0.69)
Genotypes									
ADH1B (rs1229984)									
His/His	517	0.016	(−0.00001 to 0.031)	517	0.024	(0.016–0.032)	508	−1.49	(−2.47 to −0.51)
P for interaction			reference			reference			reference
His/Arg or *Arg/Arg*	372	−0.005	(−0.025 to 0.015)	372	0.024	(0.015–0.033)	364	−1.32	(−2.44 to −0.20)
P for interaction			0.019			0.631			0.866
ALDH2 (rs671)									
Glu/Glu	482	0.001	(−0.015 to 0.017)	482	0.025	(0.017–0.032)	470	−0.64	(−1.64 to 0.36)
P for interaction			reference			reference			reference
Glu/Lys or *Lys/Lys*	407	0.002	(−0.022 to 0.025)	407	0.020	(0.009–0.032)	402	−2.57	(−3.95 to −1.18)
P for interaction			0.838			0.623			0.005
Combinations of genotypes									
ADH1B His/His + *ALDH2 Glu/Glu*	275	0.007	(−0.013 to 0.027)	275	0.024	(0.014–0.034)	270	−0.34	(−1.69 to 1.01)
P for interaction			reference			reference			reference
ADH1B His/His + *ALDH2 Glu/Lys* or *Lys/Lys*	242	0.029	(−0.003 to 0.061)	242	0.020	(0.004–0.035)	238	−3.44	(−5.25 to −1.62)
P for interaction			0.433			0.691			0.010
ADH1B His/Arg or *Arg/Arg* + *ALDH2 Glu/Glu*	207	−0.011	(−0.039 to 0.017)	207	0.027	(0.015–0.039)	200	−0.99	(−2.52 to 0.55)
P for interaction			0.161			0.555			0.556
ADH1B His/Arg or *Arg/Arg* + *ALDH2 Glu/Lys* or *Lys/Lys*	165	−0.033	(−0.069 to 0.003)	165	0.018	(−0.0003 to 0.037)	164	−0.73	(−2.99 to 1.53)
P for interaction			0.020			0.951			0.093

CI, confidence interval; HDL, high-density lipoprotein; LDL, low-density lipoprotein.

[a]Linear regression analysis adjusted for age, physical activity, body mass index, energy intake, study area, education, diabetes, smoking status, and intake of saturated fatty acids, polyunsaturated fatty acids, cholesterol and dietary fiber as covariates.

eTable 2. Associations between serum lipid levels and drinking categories according to the presence of *ADH1B* and *ALDH2* in the main study[a]

	ALL			*ADH1B* (rs1229984) *His/His*			*ADH1B* (rs1229984) *His/Arg* or *Arg/Arg*			*ALDH2* (rs671) *Glu/Glu*			*ALDH2* (rs671) *Glu/Lys* or *Lys/Lys*		
Log_e-triglycerides (mg/dL)	n	β	95%CI	n	β	95%CI	n	β	95% CI	n	β	95% CI	n	β	95% CI
Non-drinkers[b]	211		reference	126		reference	85		reference	39		reference	172		reference
Light drinkers	317	0.005	(−0.080 to 0.089)	186	−0.031	(−0.138 to 0.077)	131	0.076	(−0.066 to 0.218)	169	−0.040	(−0.215 to 0.136)	148	0.017	(−0.086 to 0.120)
Moderate drinkers	173	0.031	(−0.067 to 0.129)	103	−0.008	(−0.132 to 0.117)	70	0.114	(−0.049 to 0.277)	115	−0.058	(−0.241 to 0.124)	58	0.080	(−0.059 to 0.220)
Heavy drinkers	188	0.042	(−0.057 to 0.142)	102	0.105	(−0.024 to 0.235)	86	−0.010	(−0.172 to 0.152)	159	−0.036	(−0.216 to 0.145)	29	0.028	(−0.164 to 0.221)
P for trend			0.332			0.112			0.977			0.823			0.392
P for interaction			−			reference			0.100			reference			0.659
Log_e-HDL cholesterol (mg/dL)	n	β	95% CI	n	β	95% CI	n	β	95% CI	n	β	95% CI	n	β	95% CI
Non-drinkers	211		reference	126		reference	85		reference	39		reference	172		reference
Light drinkers	317	0.060	(0.020−0.100)	186	0.061	(0.008−0.113)	131	0.065	(0.001−0.130)	169	0.085	(0.003−0.166)	148	0.044	(−0.006 to 0.094)
Moderate drinkers	173	0.110	(0.064−0.156)	103	0.102	(0.042−0.163)	70	0.122	(0.048−0.196)	115	0.137	(0.052−0.221)	58	0.095	(0.027−0.163)
Heavy drinkers	188	0.191	(0.144−0.238)	102	0.193	(0.130−0.256)	86	0.189	(0.115−0.262)	159	0.223	(0.139−0.307)	29	0.143	(0.049−0.237)
P for trend			<0.001			<0.001			<0.001			<0.001			<0.001
P for interaction			−			reference			0.685			reference			0.295
LDL cholesterol (mg/dL)	n	β	95% CI	n	β	95% CI	n	β	95% CI	n	β	95% CI	n	β	95% CI
Non-drinkers	209		reference	125		reference	84		reference	38		reference	171		reference
Light drinkers	310	−3.24	(−8.34 to 1.85)	182	−2.19	(−8.86 to 4.47)	128	−5.89	(−13.97 to 2.19)	165	−0.71	(−11.57 to 10.15)	145	−0.92	(−7.03 to 5.19)
Moderate drinkers	171	−9.07	(−14.99 to −3.16)	103	−9.96	(−17.64 to −2.27)	68	−9.11	(−18.48 to 0.25)	113	−1.31	(−12.62 to 9.99)	58	−12.01	(−20.25 to −3.78)
Heavy drinkers	182	−11.58	(−17.60 to −5.56)	98	−12.33	(−20.40 to −4.26)	84	−11.66	(−20.91 to −2.41)	154	−5.36	(−16.55 to 5.83)	28	−16.54	(−28.03 to −5.05)
P for trend			<0.001			<0.001			0.011			0.186			0.001
P for interaction			−			reference			0.997			reference			0.016

CI, confidence interval; HDL, high-density lipoprotein; LDL, low-density lipoprotein.

[a]Performed by linear regression analysis adjusted for age, physical activity, body mass index, energy intake, study area, education, diabetes and smoking status as covariates.

[b]Drinking categories (non-drinkers [0 g], low drinkers [0.1–22.9 g], moderate drinkers [23.0–45.9 g], and heavy drinkers [≥46 g]).

eTable 3. Associations between serum lipid levels and drinking categories according to the combination of *ADH1B* and *ALDH2* genotypes in the main study[a]

	ADH1B His/His + *ALDH2 Glu/Glu*			*ADH1B His/His* + *ALDH2 Glu/Lys* or *Lys/Lys*			*ADH1B His/Arg* or *Arg/Arg* + *ALDH2 Glu/Glu*			*ADH1B His/Arg* or *Arg/Arg* + *ALDH2 Glu/Lys* or *Lys/Lys*		
Log$_e$-triglycerides (mg/dL)	n	β	95% CI	n	β	95% CI	n	β	95% CI	n	β	95% CI
Non-drinkers[b]	27	reference		99	reference		12	reference		73	reference	
Light drinkers	100	−0.152	(−0.354 to 0.050)	86	0.003	(−0.139 to 0.144)	69	0.208	(−0.124 to 0.540)	62	0.028	(−0.127 to 0.183)
Moderate drinkers	63	−0.188	(−0.400 to 0.025)	40	0.128	(−0.053 to 0.309)	52	0.248	(−0.094 to 0.590)	18	0.061	(−0.174 to 0.296)
Heavy drinkers	85	−0.049	(−0.262 to 0.164)	17	0.221	(−0.043 to 0.485)	74	0.074	(−0.260 to 0.409)	12	−0.283	(−0.581 to 0.014)
P for trend			0.706			0.063			0.530			0.338
P for interaction		reference				0.371			0.357			0.235
Log$_e$-HDL cholesterol (mg/dL)	n	β	95% CI	n	β	95% CI	n	β	95% CI	n	β	95% CI
Non-drinkers	27	reference		99	reference		12	reference		73	reference	
Light drinkers	100	0.103	(0.002–0.203)	86	0.040	(−0.029 to 0.108)	69	0.096	(−0.048 to 0.239)	62	0.057	(−0.022 to 0.136)
Moderate drinkers	63	0.147	(0.041–0.253)	40	0.061	(−0.027 to 0.148)	52	0.149	(0.001–0.297)	18	0.154	(0.034–0.274)
Heavy drinkers	85	0.224	(0.117–0.330)	17	0.156	(0.028–0.284)	74	0.249	(0.104–0.394)	12	0.130	(−0.022 to 0.282)
P for trend			<0.001			0.017			<0.001			0.008
P for interaction		reference				0.209			0.840			0.815
LDL cholesterol (mg/dL)	n	β	95% CI	n	β	95% CI	n	β	95% CI	n	β	95% CI
Non-drinkers	26	reference		99	reference		12	reference		72	reference	
Light drinkers	99	−3.82	(−17.65 to 10.01)	83	1.97	(−6.06 to 9.99)	66	1.27	(−17.18 to 19.72)	62	−5.22	(−14.93 to 4.50)
Moderate drinkers	63	−2.29	(−16.73 to 12.14)	40	−15.50	(−25.64 to −5.35)	50	−2.42	(−21.45 to 16.62)	18	−3.91	(−18.65 to 10.82)
Heavy drinkers	82	−7.22	(−21.79 to 7.36)	16	−18.90	(−34.01 to −3.79)	72	−5.19	(−23.74 to 13.37)	12	−9.73	(−28.39 to 8.94)
P for trend			0.362			0.001			0.269			0.260
P for interaction		reference				0.035			0.589			0.072

CI, confidence interval; HDL, high-density lipoprotein; LDL, low-density lipoprotein.

[a]Performed by linear regression analysis adjusted for age, physical activity, body mass index, energy intake, study area, education, diabetes and smoking status as covariates.

[b]Drinking categories (non-drinkers [0 g], low drinkers [0.1–22.9 g], moderate drinkers [23.0–45.9 g], and heavy drinkers [≥46 g]).

eTable 4. Background characteristics according to alcohol drinking habits in the replication study[a]

	All (n=2,562)	Nondrinkers[b] (n=618)	Current drinkers (n=1,944)	P^c
Age, years	52.3 (8.8)	52.0 (9.0)	52.3 (8.7)	0.470
Body mass index, kg/m^2	23.4 (2.8)	23.5 (3.0)	23.4 (2.7)	0.196
Smoking				
Current smokers, %	24.1	22.3	24.6	<0.001
Former smokers, %	43.1	34.1	46.0	
Nonsmokers, %	32.8	43.5	29.4	
Diabetes, %[d]	6.1	6.8	5.9	0.427
Education (over high school), %	54.8	54.1	55.0	0.665
Physical activity, MET･hr/day	10.5 (11.1)	10.9 (11.5)	10.4 (11.0)	0.263
Alcohol consumption, g/day	19.6 (25.1)	–	25.9 (25.8)	–
Systolic blood pressure, mm Hg[e]	117.0 (13.1)	115.8 (12.9)	117.4 (13.1)	0.015
Diastolic blood pressure, mm Hg[e]	74.1 (9.2)	73.2 (9.4)	74.5 (9.2)	0.007
Serum total cholesterol, mg/dL	202.3 (31.6)	203.6 (31.1)	201.9 (31.8)	0.236
Serum triglycerides, mg/dL[f]	123.3 (76.2)	123.6 (74.1)	123.2 (76.9)	0.528
Serum HDL cholesterol, mg/dL[f]	57.7 (15.2)	53.7 (14.0)	58.9 (15.4)	<0.001
Serum LDL cholesterol, mg/dL[g]	120.6 (29.1)	125.8 (28.9)	118.9 (29.0)	<0.001
Dietary intakes				
Energy, kcal/day	1904 (350)	1880 (335)	1911 (355)	0.059
Saturated fatty acids, g/day[f]	10.9 (2.4)	11.1 (2.7)	10.9 (2.3)	0.092
Polyunsaturated fatty acids, g/day[f]	13.2 (3.4)	13.1 (3.3)	13.3 (3.4)	0.172
Cholesterol, mg/day[f]	231.6 (64.3)	226.4 (63.1)	233.3 (64.6)	0.013
Dietary fiber, g/day[f]	10.2 (2.7)	10.3 (2.9)	10.2 (2.7)	0.899
ALDH2 (rs671)				
Glu/Glu, %	48.9	14.2	59.9	<0.001
Glu/Lys, %	43.0	55.3	39.0	
Lys/Lys, %	8.1	30.4	1.0	
Glu/Lys or *Lys/Lys,* %	51.1	85.8	40.1	

MET, metabolic equivalent; HDL, high-density lipoprotein; LDL, low-density lipoprotein.

[a]Values represent the means (standard deviation).

[b]Includes former drinkers (n=31).

[c]Performed by *t*-test or chi-square test for comparison between nondrinkers and current drinkers.

[d]The presence of diabetes was defined as a fasting blood glucose ≥126 mg/dL or hemoglobin A1c ≥ 6.5% (values of the National Glycohemoglobin Standardization Program).

[e]Participants receiving antihypertensive drugs were excluded from the analysis for blood pressure (n=435).

[f]Statistically tested for $\log_e$-transformed values.

[g]Participants with serum triglycerides ≥400 mg/dL were excluded from the analysis for LDL cholesterol (n=31).

eTable 5. Associations between serum lipid levels and drinking categories in the replication study[a]

	ALL			*ALDH2* (rs671) *Glu/Glu*			*ALDH2* (rs671) *Glu/Lys* or *Lys/Lys*		
Log_e-triglycerides (mg/dL)	n	β	95% CI	n	β	95% CI	n	β	95% CI
Non-drinkers[b]	618		reference	88		reference	530		reference
Light drinkers	1119	−0.033	(−0.080 to 0.013)	553	0.047	(−0.061 to 0.155)	566	−0.053	(−0.108 to 0.002)
Moderate drinkers	509	−0.022	(−0.078 to 0.034)	354	0.051	(−0.062 to 0.164)	155	−0.062	(−0.146 to 0.021)
Heavy drinkers	316	0.027	(−0.039 to 0.092)	258	0.070	(−0.048 to 0.189)	58	0.053	(−0.075 to 0.180)
P for trend			0.577			0.324			0.423
P for interaction			−		reference				0.996
Log_e-HDL cholesterol (mg/dL)	n	β	95% CI	n	β	95% CI	n	β	95% CI
Non-drinkers	618		reference	88		reference	530		reference
Light drinkers	1119	0.052	(0.029−0.075)	553	0.014	(−0.039 to 0.066)	566	0.044	(0.017−0.072)
Moderate drinkers	509	0.122	(0.095−0.150)	354	0.075	(0.020−0.131)	155	0.116	(0.075−0.157)
Heavy drinkers	316	0.170	(0.138−0.203)	258	0.135	(0.077−0.193)	58	0.105	(0.042−0.168)
P for trend			<0.001			<0.001			<0.001
P for interaction			−		reference				0.238
LDL cholesterol (mg/dL)	n	β	95% CI	n	β	95% CI	n	β	95% CI
Non-drinkers	612		reference	88		reference	524		reference
Light drinkers	1107	−4.37	(−7.18 to −1.56)	545	−4.15	(−10.59 to 2.29)	562	−4.53	(−7.92 to −1.14)
Moderate drinkers	504	−8.53	(−11.90 to −5.16)	353	−8.63	(−15.39 to −1.87)	151	−8.39	(−13.56 to −3.22)
Heavy drinkers	308	−12.26	(−16.24 to −8.29)	251	−11.15	(−18.28 to −4.03)	57	−17.83	(−25.72 to −9.95)
P for trend			<0.001			<0.001			<0.001
P for interaction			−		reference				0.148

CI, confidence interval; HDL, high-density lipoprotein; LDL, low-density lipoprotein.

[a]Performed by linear regression analysis adjusted for age, physical activity, body mass index, energy intake, education, diabetes and smoking status as covariates.

[b]Drinking categories (non-drinkers [0 g], low drinkers [0.1–22.9 g], moderate drinkers [23.0–45.9 g], and heavy drinkers [≥46 g]).
